# What Shall Be the Minimum Standard of Requirements for Admission to the Study and Practice of Medicine?1Presidential address delivered at the ninth annual meeting of the National Confederation of State Medical Examining and Licensing Boards, held at Columbus, Ohio, June 5, 1899.

**Published:** 1899-09

**Authors:** William Warren Potter

**Affiliations:** Buffalo, N. Y.


### WHAT SHALL BE THE MINIMUM STANDARD OF REQUIREMENTS FOR ADMISSION TO THE STUDY AND PRACTICE OF MEDICINE?[1]

BY WILLIAM WARREN POTTER, M. D., Buffalo, N. Y.

THERE is less occasion at this time for extended remark than at former meetings, when it has been my privilege to address this body. Then it was important to define our purposes, to discuss problems that were embryonic or only partially developed, and to declare our intentions as to future policy. Then the schools, the state, the people, the legislatures even, were jealous of their rights and fearful that we might invade them.

Now we meet with an accomplished task, at least in part, and with well-understood purposes as to further work that confronts us. At the present time one of the most urgent, if not the paramount, questions to consider is, What shall be the minimum standard of requirements for admission to practise upon which all the states can agree?

Confessedly there ought to be common ground upon which we all may meet as a basis upon which to build an enduring superstructure—one that shall embody all the essentials of a superior medical education.

At a period of time when the republic is taking its place in the

---

1. Presidential address delivered at the ninth annual meeting of the National Confederation of State Medical Examining and Licensing Boards, held at Columbus, Ohio, June 5, 1899.

front rank of nations, when it is putting on the vestments of strength in all that pertains to greatness in state craft, as well as in military and naval prowess, when an American admiral is receiving the homage of the world for heroic action in the far east,—for a sturdy valor that in a single day added an archipelago to our national domain and delivered 9,000,000 of people from a thraldom more oppressive than barbarism; this, I repeat, is not the time for taking backward steps in any respect, least of all in matters pertaining to medical education.

It is a matter of congratulation that since our last meeting much progress has been made in perfecting methods of teaching in the schools, as evidenced by lengthened terms and the exacting of higher preliminaries in many of the states. But it is to be regretted that here and there some lingering indications of unrest appear, and that in a few instances the states are looking if not stepping backward.

It is not my desire to particularise, but I am sure that all will agree that the recent amendment, adopted by the legislature of Tennessee, exempting the graduates of schools within its borders from examination is to be regretted. In New York, too, a bill passed both houses of the legislature last winter exempting those medical officers that served in the war with Spain from the preliminary requirements that apply to others. But our sturdy common sense governor declined to approve of it, thus saving us the humiliation of weakening our wholesome statute.

In a former discourse I had occasion to speak of the alertness necessary to be exercised by the friends of state control to prevent the passage of insidious amendments to existing laws. Interested persons, designing men, adventurers and lobby fiends are lurking about legislative halls, with a constant design to undo or weaken the bulwarks that have been erected against ignorance and quackery. Now, more than ever, vigilance seems to be required to prevent the machinations of these aberrant forces that, singly or combined, would work havoc with our system. There is at present a tendency to overlegislate, and it is becoming almost more difficult to prevent too much or too dangerous legislation, than to obtain adequate or needful action by legislators. It has always been a great mystery to me why it is apparently easier to get the ear of a legislative body for improper or needless work, than for such as would improve the body politic; why an inconsequential subject or an irresponsible person seems to possess greater attraction for the average "member," than well-considered bills offered by persons who are entitled to credence, or to those that spring from official sources ! !

It is getting to be the work of humiliation for a busy physician in respectable standing to approach his "member" in the interest of the people. He frequently gets a curt answer or an intimation that the aforesaid member is very busy with his own or other affairs. To approach a legislative body in session is an expensive affair, involving loss of much time, costly and dangerous travel, and the payment of princely hotel bills. Doctors are not likely to meet such conditions with grace or unruffled feelings, hence they let many things go by default rather than be subjected to such humiliation. These are perhaps some of the reasons why we are confronted in some of the states with retrogressive legislation.

Do not misunderstand me: there are very many good conscientious public men—industrious and honest legislators—who would be the last men to do individual wrong to a constituent, no matter how humble; but with a legislature as a whole when in session, with its never-ending list of rules, its parliamentary tactics, and its mysterious committees, it becomes quite another thing. The individual member is lost sight of in the grouping of the whole, and his vision is less acute or his sensitiveness more obtunded than when at home. This is not said in a spirit of criticism or disappointment, for, by and large, perhaps personally I have fared better than I deserved, when I have had the temerity to approach either single members or legislative bodies in session. In these remarks I have merely endeavored to point out an evil that confronts us, and to make of it an argument for united and forceful effort to hold the ground we have occupied as well as into press forward into new territory. In other words, let us permit no retrogressive legislation in those states that already have wholesome laws controlling medical licensure ; furthermore, let us do our utmost to stimulate the enactment of similar statutes in states and territories where none, or at best, inadequate laws exist.

This brings me again to the consideration, for a moment, of the recognition by each state of licenses granted by others. Undoubtedly, it would be most desirable if all the state licenses were interchangeable or interindorsable, for then a license of any state could roam all over the country with a free lance. It so happens, however, though twenty-eight or more states grant licenses, that no two have, in all respects, equal standards. Some of them merely visé a legal diploma; others examine only candidates that have no diplomas; still others require no preliminary education; while a few grant licenses only to those who give proof of preliminary education, three or four years of medical college teaching, and a diploma.

As a remedy it has been proposed in certain quarters to abolish state examining boards and establish in their stead a national board; but a moment's thought will convince any reflecting mind of the impracticability, not to say impossibility, of carrying out such a scheme. The states will not surrender the right to make their own police regulations, nor will the general government deprive the states of their vested constitutional privileges.

The real remedy consists in establishing uniform standards of requirements in all the states. This should embrace the triad—preliminary education, medical collegiate training, and state examination for license. It would not, or at least ought not to, be difficult for the states to determine that a high school diploma, or its equivalent, shall be the minimum of preliminary requirement; and that without this the schools shall not receive students. Colleges should then be required to establish four years' courses and finally, a state examination of uniform standard in all the states, no candidate to be admitted to it unless he presents a diploma from a school recognised as maintaining the proper preliminaries for admission.

A national clearing house made up of representatives from the several states could pass upon or visé the diplomas of such as desire to change states—such visé to entitle a diploma to registration in the state in which its holder desires to take up residence. While there are many opinions on this vexed question, most of which are made up from special cases of so-called hardship, there really remains but one equitable solution of the problem, and that is for the several states to establish uniform standards of preliminaries, collegiate courses, and state examinations.

The character of the examination imposed by the state has been the subject of criticism. One critic would ask simpler questions; another would make them more severe; still another says the questions are too bookish, or are not practical in character; yet another would institute a clinical or bedside examination in the hospital wards; while a fifth would have us divide the examination, making an undergraduate test at the end of two years in anatomy, physiology and chemistry. It is easy to find fault, but difficult to attain the ideal. The fourth proposition is well-nigh absurd because, first, it cannot be carried out; and, second, it is unnecessary. We are examiners not teachers, and our province is simply to determine the nature and value of the instruction candidates have already received and whether it adequately fits them to practise. These latter reasons will apply equally well to the proposition to divide the examinations.

Heretofore, an American diploma has had no standing abroad, and the holder could obtain no privileges under it; whereas, the possessor of a foreign diploma could come to our shores and practise with a free lance, not even saying 'by your leave?' Under the present system all this will be changed. A New York or an Ohio license will be recognised in Europe else a European license will not be recognised in New York and Ohio.

I am well aware that the propriety of establishing so high a standard of preliminaries as proposed has been questioned in some quarters, notably in the southwest. It has been there suggested that in the mountainous regions of some of the states a high order of education is not required; that if it is established the inhabitants there will be deprived of medical aid because educated men will not settle among the mountaineers; that medical colleges cannot obtain support with such standards at their thresholds, and that for these and similar reasons a lower standard should be permitted. I am sure no one in this presence will admit the force of such arguments. For instance, shall the State of Ohio undo its great work of the past and let down the bars to ignorance and greed? When states are modeling legislation after that already established by some and are even creating systems of a higher range, it would appear unwise to "crook the pregnant hinges of the (our) knee, where thrift may follow fawning," to gratify a few who wonder if it is proper to shut the doors against those of inadequate early training.

Come with me for a moment into the regents' office in the Capitol at Albany and let us examine the records. Among the examination papers there on file we will find the following exhibition of gems:

Funic shueful for funic souffle.

Placental shuffle for placental souffle.

Umbilicle scoffle for umbilical souffle.

Umbilikes and umberlicus for umbilicus.

Apetate for appetite.

Sluffing for sloughing.

Coxix for coccyx.

Buttix and buttex for buttocks.

Dessigna seretena for decidua serotina.

Cureate for curette.

Profolaxis for prophylaxis.

Insission for incision.
Sillia for cilia.
Fetted for fetid.
Disisses for diseases.
Physition and phesicean for physician.
Oxiput for occiput.

How would any person in this presence enjoy the professional services of one of these "physitions?" These are only a few specimens of illiteracy to be found in the regents' medical archives; they are taken at random from a few recent examination papers of candidates who, let it be remembered, hold college diplomas, as otherwise they would not have been admitted to the examinations. Moreover, they may be regarded in a certain sense as the selects or cream of the applicants for license, as the practice law has reduced the number of candidates who annually apply, to a number very much less than that which was registered each year under the old system.

I have purposely omitted all words from this list not pertaining to medicine. Had an attempt been made to give illustrations of strictly English orthography, I fear you would have deemed the record almost apocryphal, if nothing worse.

It is deemed essential everywhere that a physician shall be a gentleman. How can he obtain this dignified position and be entitled to be so classified unless he is educated at least to the extent contemplated by the report of your Committee on Minimum Standards?

A leading medical journal[1] has lately presented in its editorial columns a timely setting forth of several phases of this important subject. I cannot bring this address to conclusion more profitably than by quoting a few paragraphs therefrom. This writer says:

Those whose task it has been to read and mark medical examination papers must have often noted the lack of literary ability displayed by some students who have a fairly good comprehension of the subject. These are papers where to award one due merit is a severe trial.

It is a question whether great illiteracy—though coupled with a fair knowledge of the subject—should not at once doom the candidate to rejection. A junior or senior should, we think be sent back to the study of his spelling-book, grammar and dictionary, who in answer to the question: "What is an abscess?" replies, "An abscess is a colecshun of puss in a circumscribed cavity." A candidate for the degree of M. D., who spells inflammation with one m; suppuration, supperation; capillaries, cappilaries; bacillus, baccilus; and even blunders just as badly with common English words, and who can hardly write a sentence grammatically, cannot be expected to honor the learned profession to which the diploma of a medical school gives him admission.

1. Boston Medical and Surgical Journal, June 16, 1898.

It may be said that these are very exceptional instances, but there are examiners in some medical schools who would be forced to admit that they are too common. It is from just this illiteracy and ignorance that our leading schools of medicine have obtained or are trying to obtain emancipation. It cannot but be admitted that the standard of medical education is this country is advancing. This is first of all apparent in the greater thoroughness of the matriculation examinations. The grossly illiterate must and will be excluded. Those medical schools (and they are too few) that propose to make matriculation dependent upon the possession of a degree of arts, (or its equivalent) have taken a step in the right direction. Moreover, through affiliation with the Association of American Medical Colleges, many schools have raised the grade of requirements, both of matriculation, and of the entire curriculum; and there is always a noticeable feature of progress in the fact that a four-year course of study is being very generally adopted.

Concluding these somewhat rambling remarks, permit me to suggest that it would seem apparent from what I have said that the proposition with which I started—namely, that the most important question with which we are confronted assuredly is, What shall be the minimum standard of requirements for admission to the study and practice of medicine?

I trust the answer will be made in the adoption of the report of the committee to be presented today for the action of this body.[1]

284 FRANKLIN STREET.

